# “Employability in context”: graduate employability attributes expected by employers in regional Vietnam and implications for career guidance

**DOI:** 10.1007/s10775-022-09560-0

**Published:** 2022-07-27

**Authors:** Ly Thi Tran, Nga Thi Hang Ngo, Hoa Thi Mai Nguyen, Truc Thi Thanh Le, Tien Thi Hanh Ho

**Affiliations:** 1 Research for Educational Impact (REDI) and School of Education, Deakin University, Geelong, Australia; 2 Tay Bac University, Quyết Tâm, Tây Bắc, Vietnam; 3 School of Education, University of New South Wales, Sydney, Australia; 4 Phu Xuan University, Hue, Vietnam

**Keywords:** Employability attributes, Employers, Career guidance, Graduate employability, Higher education, Vietnam, Regional labour market

## Abstract

This article examines how graduate employability is viewed by employers in six economically disadvantaged mountainous provinces in Vietnam. The study reported in this article identified continuous self-learning, resilience, adaptability, devotion and empathy for the local people and local community to be among the main employability attributes expected of graduates in regional Vietnam. The findings of the study raise the importance of context situatedness in looking at employability and show how employability is characterised by the local structural conditions, demographic features and socio-cultural norms. The study provides significant implications for career guidance and graduate employability development, especially in relation to regional areas.

## Résumé

**"L'employabilité dans son contexte »: Attributs attendus par les employeurs·euses dans la région du Vietnam et implications pour l'orientation professionnelle** Cet article examine comment l'employabilité des diplômé·e·s est perçue par les employeurs·ses dans six provinces montagneuses économiquement défavorisées du Vietnam. L'étude rapportée dans cet article a identifié l'auto-apprentissage continu, la résilience, l'adaptabilité, le dévouement et l'empathie pour la population locale et la communauté locale comme étant parmi les principaux attributs d'employabilité attendus des diplômés dans le Vietnam régional. Les résultats de l'étude soulignent l'importance de la situation du contexte dans l'étude de l'employabilité et montrent comment l'employabilité est caractérisée par les conditions structurelles locales, les caractéristiques démographiques et les normes socioculturelles. L'étude fournit des implications significatives pour l'orientation professionnelle et le développement de l'employabilité, en particulier en ce qui concerne les zones régionales.

## Abstract (de)

**"Arbeitsmarktfähigkeit im Kontext": Von den Arbeitgebenden in der Region Vietnam erwartete Eigenschaften und Auswirkungen auf die Berufsberatung** In diesem Artikel wird untersucht, wie die Arbeitgebende in sechs wirtschaftlich benachteiligten Bergprovinzen Vietnams die Arbeitsmarktfähigkeit von Hochschulabsolvent*innen einschätzen. Die Studie hat ergeben, dass kontinuierliches Selbstlernen, Resilienz, Adaptabilität, Hingabe und Empathie für die lokale Bevölkerung und die örtliche Gemeinschaft zu den wichtigsten Eigenschaften der Arbeitsmarktfähigkeit gehören, die von Hochschulabsolvent*innen im regionalen Vietnam erwartet werden. Die Ergebnisse der Studie verdeutlichen die Bedeutung der Kontextbezogenheit bei der Betrachtung der Arbeitsmarktfähigkeit und zeigen, wie die Arbeitsmarktfähigkeit durch die lokalen strukturellen Bedingungen, demografischen Merkmale und soziokulturellen Normen geprägt wird. Die Studie liefert wichtige Implikationen für die Berufsberatung und die Entwicklung der Arbeitsmarktfähigkeit, insbesondere in Bezug auf regionale Gebiete.

## Resumen

**"Empleabilidad en contexto": Atributos esperados por los empleadores en Vietnam regional e implicaciones para la orientación profesional** Este artículo examina cómo los empleadores ven la empleabilidad de los graduados en seis provincias montañosas económicamente desfavorecidas de Vietnam. El estudio reportado en este artículo identificó el autoaprendizaje continuo, la resiliencia, la adaptabilidad, la devoción y la empatía por la población local y la comunidad local como uno de los principales atributos de empleabilidad que se esperan de los graduados en la región de Vietnam. Los hallazgos del estudio plantean la importancia de la situación contextual para observar la empleabilidad y muestran cómo la empleabilidad se caracteriza por las condiciones estructurales locales, las características demográficas y las normas socioculturales. El estudio proporciona implicaciones significativas para la orientación profesional y el desarrollo de la empleabilidad, especialmente en relación con las áreas regionales.

## Introduction

The development of graduate employability has increasingly become a priority for universities and nation states around the world since it is regarded as a powerful vehicle to improve the quality of human capital. In Vietnam, concerns about graduate employability have been consistently raised over the past two decades because of rising graduate unemployment, inadequate work readiness and employers’ unsatisfaction about graduate skills development. These concerns have been accelerated due to changing demands and structures of the contemporary labour market, characterised by a rapid increase in international trade and private, joint venture and foreign direct investment (FDI) enterprises (Anwar & Nguyen, 2014; Nghia, 2019; Nghia & Tran, 2020; Nguyen et al., 2019; Tran et al., 2018a, 2018b). There has been a significant growing demand for graduates with capabilities to engage and perform effectively in a socialist-oriented multi-sectoral market economy. In addition, the accelerating demand for regional and global engagement and competition for Vietnam also means the country needs more qualified human resources with a good command of English capable to work not only in a more ‘internationalized’ local labour market but also in the region and the world (Tran & Marginson, 2018; Tran & Nørlund, 2015; Tran et al., 2018a, 2018b). In response to the critical need to enhance graduate employability in accordance with new demands of the labour market and to improve the quality of higher education in general, major higher education reforms have been implemented since early 2000s (Bui et al., 2019). It is a critical time to revisit graduate employability in Vietnam, especially given that the COVID-19 pandemic has exacerbated concerns about rising unemployment, heightened job competition and economic recessions.

In an attempt to understand the skills, knowledge and attributes needed for the current labour market in Vietnam, a small but growing body of research has examined the demands and gaps with regard to graduate employability from the perspectives of employers, graduates and students (Nghia, 2019; Nguyen, 2011; Tran, 2015; Tran & Bui, 2021; Tran & Swierczek, 2009; Tran et al., 2021). While this growing body of research is a welcome development in the existing literature on graduate employability in Vietnam, it has largely concentrated on the perspectives of employers, graduates and students in urban areas, especially in big cities such as Hà Nội and Hồ Chí Minh City (e.g. Ly et al., 2015; Tran, 2015; Tran & Swierczek, 2009). Even though concerns have been raised with regard to the equity and quality disparity in universities across different regions in Vietnam (Tran et al., 2014), little is known about the extent to which employers in the disadvantaged regions of the nation are influencing and creating demands for universities in this region and what employability skills they expect out of graduates. Furthermore, Vietnam is the world’s 15th and ASEAN’s 3rd largest nation with a population of over 98 million people and up to 63 per cent of the population are living in rural areas (World Bank, 2019). Yet, there has been a dearth of research on identifying the employers’ needs and graduates’ skills gaps in regional Vietnam. This study is the first that aims to capture the voices and needs of employers regarding graduate employability in the economically disadvantaged Northern mountainous region of Vietnam, which is populated by many ethnic minorities.

### Graduate employability development

What constitutes graduate employability is controversial and varies across different contexts. Scholars in the field highlight the need to differentiate between ‘graduate employability’ and ‘graduate employment’, which refers to holding a job (Bennett, 2020; Yorke, 2006), and emphasise that the relationship between employability and employment is not straightforward (Wilton, 2011). Yorke (2006) refers to employability as:

a set of achievements – skills, understanding and personal attributes – that makes graduates more likely to gain employment and be successful in their chosen occupations, which benefits themselves, the workforce, the community and the economy (p. 23).

In many studies, employability is seen to include technical employability skills and generic employability skills (Bowman, 2010). Bennett (2020) offers a broader definition of employability, which underscores the ability “to find, create and sustain meaningful work across the career lifespan” (p. 5). Graduate employability can be conceptualised as including four main components: human capital, social capital, individual behaviours and individual attributes (Clarke, 2018). These dimensions seem to be interrelated and depend largely on graduates’ habitus developed and modified by their individual circumstances as well as the education and socio-economic conditions which they have access and are exposed to. Scholars also argue for the importance to view employability from a broader and systemic integrative approach, taking into account educational and governmental, organisational and individual perspectives (Guilbert et al., 2016). Importantly, graduate employability depends on the supply and demand on the labour market, the interaction between employers, industry, education providers, individual students and related communities (Tran et al., 2014). It cannot be seen as a stable set of skills across contexts. This echoes the need for further investigation of employability skills in different contexts from different stakeholders, including employers.

In Vietnam, generic employability skills are often referred to as soft skills. These skills include communication, teamwork, problem-solving and critical thinking skills and creativity, among others (Hager & Holland, 2006). Various studies, which concentrate on perspectives of graduates and employers from the urban areas, consistently indicate Vietnamese graduates lack soft skills and work readiness (e.g. Ly et al., 2015; Mai, 2018; Nghia, 2019; Pham & Tran, 2013; Tran, 2015; Tran & Swierczek, 2009). Research into graduate employability in Vietnam has identified different sets of skills and attributes demanded by employers over different socio-economic periods of the country. Prior to Đổi Mới in 1986 which marked the nation’s shift from a centralised and subsidised economy to a market one, qualities such as obedience, loyalty, industriousness, diligence and the ability to follow orders appeared to be valued in the subsidised, centralised economy (Nguyen, 2009). As the nation moves towards a multiple-sector, socialist- and market-oriented economy with state-controlled creativity after Đổi Mới, it demands a workforce with the ability to work independently and creatively, and to cope with pressure (Tran & Swierczek, 2009). In the current context of Vietnam’s labour market, characterised by the advancement of technology, workforce re-structuring and dramatic changes in socio-economic conditions, skills such as communication, teamwork, life-long learning, critical thinking, problem solving and foreign language competence appear to be important skills required by employers (Mai, 2018; Tran et al., 2014). Despite the awareness of these skills gaps faced by the employers, there remains a lack of systemic understandings of skills needs at the company, sectoral and regional levels (DFAT, 2014). Existing literature also suggests a lack of partnerships between industry and universities with limited involvement of employers in the development and delivery of university programmes (DFAT, 2014).

#### The context of Northern mountainous region of Vietnam

Vietnam’s Northern mountainous region consists of 14 provinces, namely Hòa Bình, Sơn La, Lai Châu, Yên Bái, Lào Cai, Điện Biên, Hà Giang, Cao Bằng, Bắc Cạn, Lạng Sơn, Thái Nguyên, Bắc Giang, Phú Thọ and Tuyên Quang (Vietnamese Government, 2010). This region is the largest of the six regions in Vietnam, with a total area of approximately 95,223 km2, accounting for 28.7% of the country’s surface (GSO, 2016). According to the latest data by the General Statistics Office of Vietnam, the Northern mountainous region has a population of 12.726 million people, which is almost 13% of the country’s population (GSO, 2020). Educationally, the northern mountainous region experiences several challenges due to its segregated, mountainous topography, large concentration of ethnic minorities, high proportion of households living below poverty line, shortage of teachers who are ethnic minorities or speak minority languages, and lack of employment prospects (e.g. ISEE, 2011; Tran et al., 2015).

There are four universities in the Northern mountainous region, namely Thái Nguyen University (which includes seven member universities), Tây Bắc University, Tân Trào University and Phú Thọ University. Among them, Thái Nguyên University is one of the key regional multi-discipline, multi-major and multi-mode universities in Vietnam (Thái Nguyên University website). Apart from the universities, there is a network of colleges and vocational schools in the region.

The General Statistics Office data show that in 2019, there were over 7.7 million of working people aged 15 and above, accounting for almost 61% of the total population of the Northern mountainous region (GSO, 2020). The proportion of trained workers was only 17.5%. Despite the various efforts and investments by the government, the proportion of students attending vocational training after graduation from lower and upper secondary schools remains low (Tran et al., 2010). According to Tran et al. (2010), one key inhibitor is the limited employment opportunities. In case studies in different sites in the Northern mountainous region, the researchers highlighted the mismatch between training programmes and labour needs. Since the training programmes were not based on research on local or employers’ needs, graduates struggled to find jobs upon graduation. A large proportion of them could not manage to gain employment and had to return to low-skill jobs that did not require such training so as to earn a living; others ended up in debt since they could not pay back the loans they took to participate in these programmes. Consequently, people were not interested in vocational training programmes since they lost trust in the effectiveness of such training (ISEE, 2011).

## Positioning theory as a conceptual framework to understand the ways employers position employability

In this study, positioning theory is used to interpret the ways employers position the employability capabilities expected of graduates and the skills gaps they encounter. Positioning theory is concerned with “the discursive processes by which people were ascribed, took up, refused, contested, and so on the rights and duties they found themselves with in the local social world” (Harré, 2012, p. 195). There are three core components that form an interactive triangle within positioning theory: speech acts, positions and storylines, that reveal how people position themselves and others in an interactive condition (van Langenhove & Harré, 1999). Barnes (2004, p. 3) points out that how people position themselves and are positioned by others is subject to “the context and community values and on the personal characteristics of all the individuals concerned, their personal history, their preferences and their capabilities”. Traditions and customs (Moghaddam et al., 2008) and rules and habits (van Langenhove, 2017) play a key role in shaping what people can and will do in a given situation. Positioning theory highlights different ways the norms in the society are constructed as some are universal while others are subject to understandings of rights and responsibilities which can be locally defined (van Langenhove, 2017).

Positioning theory refers to how conversations can reveal the discursive construction of personal stories through which a person’s actions can be made intelligible and relatively determinate as social acts (van Langenhove & Harré, 1999). Therefore, in this study, employers’ conversations about their expectations of graduates can reveal the norms governing the local labour market as well as the structural conditions that determine graduate employability. Through reflective and interactive positioning, people allocate certain positions to themselves, which is referred to as deliberate self-positioning, and to others, which is referred to as other positioning (van Langenhove & Harré, 1999). Each position is often associated with a set of rights, duties and/or obligations (Kayi-Aydar & Miller, 2018). In this study, students and graduates were positioned at the centre of the discussions in relation to other stakeholders that place a key role in their career education and employability development, including lecturers, university leaders, employers, family and local community. The interviewees self-positioned and other positioned students, graduates and their employability in relation to the structural conditions such as the labour market, the local context, the university setting, traditions and cultures. In this regard, positioning theory is used to explain how employers’ self-positioning and other positioning reveal their interpretations of the corresponding expectations, rights and responsibilities relative to these positions during the interviews.

## Method

### Participants

This study funded by Aus4Skills (see Tran et al., 2018) sought to interpret the ways employers position graduates and the employability attributes expected of them. Forty employers across six provinces in Vietnam’s Northern mountainous region including Sơn La, Hòa Bình, Điện Biên, Lào Cai, Yên Bái and Thái Nguyên, were recruited for the semi-structured interviews through a snowball approach. Consideration was made to ensure that the selected participants had knowledge and nuanced insights in graduate recruitment in their field through one of the key criteria for selection that participants should be leader of the organisation, HR manager or HR staff with experience in the sector. We ensured the inclusion of representatives from: small, medium and large companies; public and private sectors; major sectors in specific provinces. We also ensured representatives were balanced across the provinces in the Northern mountainous region. Participants’ identity and organisations are kept anonymous. Table 1 provides demographic information of the employer participants.

**Table 1:** Demographic information of employer participants (*N* = 40)

Demographic information of employer participants	No. of interviews
Gender	
Male	19
Female	21
Age group	
22–25	1
26–30	9
31–35	3
36–40	12
41 +	15
Education	
Bachelor	31
Master	9
Position	
HR staff	13
HR manager	4
Organisation leader	23
Years of experience in the current position	
0–5	23
6–10	11
11–15	6
Major sector	
Electronics	12
Agriculture	4
Telecommunication	3
Education	7
Construction	4
Education and construction	2
Hospitality	1
Other	7
Sector	
Private	8
Public	20
Cooperation	12

### Measure and procedure

In line with the semi-structured interview approach, some guided questions were used, but there was flexibility for the researchers to ask follow-up questions, to elicit interviewees’ articulation of why a specific attribute is needed. The researchers asked a key question related to a specific theme and the employers’ responses provided the following impromptu questions. The interview guide includes questions related to three main areas: (1) employers’ expectations of graduates’ knowledge, skills and attributes; (2) employers’ assessment of graduate employability; and (3) university and industry collaboration in enhancing graduate employability. This article focuses mainly on the attributes that employers expected of graduates. Ethics application was submitted and approved by the university where the lead researcher was based. All the participants signed a consent form prior to their interviews.

The interviews with employers were conducted in 2018 by two researchers of this project, who are also authors of this article. Each in-depth face-to-face interview lasted from 30 to 60 min. All interviews were conducted in Vietnamese and translated into English by the research team. The researchers are native speakers of Vietnamese and have a good command of English due to their post-graduate education in English speaking countries. They had conducted several education projects in the Northern mountainous region of Vietnam. The researchers provided both insider and outsider insights as they are familiar with the local context but had not had experience in interviewing employers in the given context prior to this project.

### Data analysis

In this study, qualitative data were analysed using content analysis (Corbin & Strauss, 2008). The data analysis method starts with a line-by-line analysis of the transcribed text to describe the context in which issues occurred (Corbin & Strauss, 2008). The transcripts of the 40 interviews were coded inductively. The two researchers first worked independently and then together on one transcript using the methods of analysis described previously. The first attempt at coding yielded an inter-rater agreement of 70%. The differences were discussed. All discrepancies in coding were reviewed, and a general consensus was reached by both researchers, ultimately resulting in 95% agreement.

One of the researchers sorted different codes into potential categories and collated all the relevant coded extracts within the categories. The researcher sorted the different categories into various themes and subthemes during this stage of the analysis process. She used mind maps to organise the theme piles concurrently and to consider the relationships between the themes and between the levels of the themes, including the drawing up the main overarching themes and subthemes within them. The analysis allowed the themes to emerge from the participants’ words rather than beginning with a hypothesis or theory that needed to be substantiated. The themes were then analysed from the lens of positioning theory as discussed above. The analysis of findings addresses how employers in this study position graduate employability attributes needed in the labour market and skills gaps entered by themselves.

## Results

The following section will present findings about the expected employability attributes that are relatively distinctive to the context of Vietnamese Northern mountainous region. These include empathy, commitment and devotion, adaptability and flexibility, ability to cope with pressure and resilience, self-learning and life-long learning and foreign and minority languages competences.

### Empathy

One of the important individual qualities critically needed and highly valued by many Vietnamese employers in the Northern mountainous region is empathy. This is related to graduates’ ability to understand and share the feelings and challenges of the locals. In particular, agriculture graduates’ empathy with local farmers, ‘winning the hearts and minds of farmers’, was considered a necessary attribute to get along with people at the grassroots level and assist them with agriculture development, which is a core responsibility of their job (Toàn and Nam, agriculture). This empathy could be demonstrated in a willingness to share challenges and difficulties with farmers through the trio of ‘living, eating, and working with farmers’ [‘*Ba cùng: cùng ăn, cùng ở, cùng làm với người dân*’] (Toàn). Similarly, in the forestry sector, the willingness to be close to and collaborate with local people who rely on rudimentary logging and hunting practices was an essential characteristic of a devoted forestry officer. For education students, the love for the teaching profession and empathy with students, mostly ethnic minority ones, being disadvantaged by geographical locations, linguistic competence and poverty, was greatly valued (Bình, education). However, Bình admitted that there was an irony in this expectation because the existing situation of unemployment in the education field adversely affects students’ morale and motivation for the job. It is very difficult for education graduates to secure a job as a teacher upon graduation, and many of them have to find an alternative pathway.

The analysis of employers’ positioning of graduates’ employability skills and attitudes shows that empathy is a critical factor that optimises employees’ successful interaction and inter-personal as well as professional relationships with the local people with whom they work closely. Empathy unveils how employability is context situated, and in this case, it is closely shaped by the demographic features of the Northern mountainous region where a large proportion of the locals are minority groups living in low social-economic situations. Within positioning theory (Harré, 2012), the storylines revealed by the employer participants show that a meaningful interactive condition between graduates as employees and locals in the Northern mountainous region is crucial to ensure their effective engagement and performance at work. In light of positioning theory, positioning often occurs in a certain context characterised by sets of rights and duties situated in socio-culturally specific systems that can both enable or hinder what can be done in a given situation (van Langenhove, 2017). In the demographic setting of the Northern mountainous region of Vietnam, empathy as a form of ‘employability in context’ appears to be a desirable attribute or duty expected of graduates when dealing with local people, of whom the majority are from disadvantaged minority groups.

### Devotion and commitment

The interview data suggested that candidates’ devotion to and passion for jobs was regarded as crucial components of ‘employability in context’ by most employers in the Northern mountainous region. In particular, employers positioning of employees’ devotion to and enthusiasm for work was discipline based and context bound. Newly graduated construction engineers, for example, were expected to be willing to work far from home in mountainous areas under unfavourable living conditions, severe weather and with difficult travelling (Lan, construction).

Additionally, responsibility and commitment are regarded as required attributes of employees working for a telecommunication company. Trinh, an employer in telecommunication, elaborated on these requirements:

Staff are not very responsible. For example, in the urgent situation when the machine is broken and needed to be fixed as soon as possible, they won’t do it because it is not their shift or working time. They think it is the company’s responsibility, not theirs. That shows their low commitment and irresponsibility. What not only my HLT company but also other companies expect is the staff’s commitment. They need to work hard and be committed to the company’s development. (Trinh, telecommunication)

One of the challenges that companies in the mountainous areas face is the instability of staff. Due to the severe weather and remote geographic location, there are only a small number of staff staying with the companies for a long time. Therefore, commitment is highly valued by the companies in this region: ‘As when I started working for this company, my boss said that the company needs staff who have high commitment with company’ (Lan Anh, electronics).

Graduates’ passion for the job was particularly associated with their aspiration to contribute to the organisation despite initial difficulties at the set-up stage (Trinh, telecommunication). During probation time, this attribute could also be revealed when employees ‘prioritised work productivity’ and ‘were willing to work overtime to finish work’ and ‘were able to resolve unexpected problems beyond their working hours’ (Trinh, telecommunication). Trinh added that many new graduates appeared to feel satisfied with their offered salaries and did not seem to make efforts in their jobs after being recruited. Their lack of aspirations and ambitions was likely to originate from ‘their wish to have a stable job and life’ (Trinh). Obtaining job stability and security or long-term/life-long employment is a traditional conceptualisation of the purpose of employment in Vietnamese culture. This is more pronounced in the Northern mountainous region where job opportunities are limited. However, given the currently fast changing and increasingly competitive labour market, it is essential for graduates to be aware that employees’ passion for their jobs and their yearning to make contributions is a desired attribute that most employers want in graduates to develop their organisations further, as highlighted by the participants quoted above.

The term ‘commitment’ in this study was referred to by participants as graduates’ ability to work for an organisation for a long duration. While it was unsurprising that employers demanded graduates’ long-term commitment to their organisations, it was interesting that this attribute was found to be strongly linked to candidates’ living areas. Many participants whose organisations were based in Thai Nguyen reported mainly hiring employees who had their permanent residency in Thai Nguyen or neighbouring areas. Some considered it to be the first criterion of recruitment to ensure employees’ loyalty to their organisations. This perception is reflected in the following quote from Hồng, an HR personnel:

Oh yes, most graduates employed by our company had their family register in Thai Nguyen. Most of them. A criterion of our company is not to recruit those from the centre or the north because they will not be committed to our company in the long term. (Hồng, electronic technology)

Sharing Hồng’s perspective, another employer, Việt Anh, believed that hiring local people increased the possibility of staff’s longer commitment because an organisation did not want to waste its time and resources to train new employees who were more likely to leave for more exciting opportunities in other regions after a short period of time. These findings about recruitment practices that tend to favour local residents due to their perceived stronger loyalty imply that graduates would need to consider and demonstrate to employers their willingness to have long-term commitment to the organisation to enhance their employability.

Within positioning theory, it can be seen that there are multiple forces involving not only the labour supply and demand, the professional field of work and workplace culture, but also the distinctive demographic conditions of the region. These interrelated forces represent the discursive processes by which employees were ascribed with expected duties (Harré, 2012, p. 195). In the context of the mountainous region, these ascribed duties explained by employers include devotion and commitment. In the discourse of graduate employability development, these become the attributes expected of graduates in the local social world.

### Adaptability and flexibility

In light of positioning theory, traditions, norms and rules (van Langenhove, 2017) are among the structural factors that determine expectations of individual duties and actions. The findings indicated that there was a paradox in how graduates are positioned in the current system of recruitment and usage of labour force in Vietnam. A proportion of graduates were assigned to work in fields different from what they had been trained and to undertake multiple tasks at a time. This is evidenced in Mai’s statement below:

[…] we want to recruit a person who can work in multiple positions and roles. For example this time he can work in this position but later on another position requires him we can move him around. It’s his ability to adapt. This department needs this role or that department needs that role, we can adjust and ensure that employees can adapt. (Mai, electronic manufacturing)

Similarly, Trinh, a participant working for a telecommunication company, reported that she had been originally employed to work on environmental issues for her enterprise; however, she was later required to take professional duties of an HR officer because her specialised area did not involve much work. An interpreter after recruitment might be required not only to do interpreting work but also to undertake other responsibilities such as making reports (Huệ, electronic manufacturing). Employees, as this study suggests, are positioned by Vietnamese employers to get ready for new tasks and roles after they are recruited, which obviously requires them to be highly flexible and adaptive. Adaptability can help the employees overcome difficulties and stay with their job. This point of view is clearly articulated in the following quote:

Ability to adapt in a new working environment is very important, particularly to my company. Because, as you know, my company is restructuring and equitizing, staff need to adapt to the new positions and changes of the company. Our company has prepared for restructuring the company since 2015 and now the staff must change their work skills. Some staff cannot adapt, they felt so tired and exhausted that they quit the job. Some old staff felt a little bit behind. Adaptability is the skill that is particularly required at a specific time. Adaptability is very vital. (Thuy, communication)

The positioning of adaptability and flexibility as important qualities required of graduates in the Northern mountainous region of Vietnam aligns with Clarke’s (2018) framework of graduate employability in which these are also regarded as core individual attributes.

In the field of construction, an engineer was expected to be able to work in a variety of working conditions such as in the office or at the construction site. In the field of production, a graduate might be demanded to work in an environment of ‘complete sanity’ where they had to ‘wear cover clothes from top to toes all day long’ (Trinh, telecommunication). Similarly, the growing competition in the banking sector required flexible responses (Sơn, banking). Especially the diverse changing economy demanded employees in banking to build up the capacity to ‘sense’ and respond flexibly (Sơn). In interior design, salespeople need not only sales and communication capabilities but also the skills to synthesise, provide advice in relation to finance and design and architecture. To develop these skills and knowledge requires graduates to be adaptable, flexibly undertake various roles in their jobs and continuously research and undertake self-study. This is echoed by previous research which highlights the crucial need for Vietnamese graduates to build capacity so as to engage in employment mobility across different sectors and tackle new employment challenges (Tran et al., 2014) given the changing multi-sectoral market-oriented economy accompanied with dynamic social and employment structures, new professions or transformed professions.

What is distinctive about the way adaptability is perceived by employers in the region is subject to its demographic mountainous conditions in which adaptability as a form of ‘employability in context’ is interlinked with graduates’ ability to change and respond effectively to constant challenges in the harsh working environment. In the forestry sector, for example, flexible adaptation and response to unpredictable situations was expected from candidates because the nature of the job in this remote mountainous region required them to analyse and respond flexibly to a range of circumstances such as deforestation, wood smuggling, or illegal exploitation. The skill to flexibly collaborate with local people to analyse and judge specific situations encountered was especially crucial (Việt, forestry).

#### Ability to cope with pressure and resilience

Employers in the Northern mountainous region in this research valued applicants’ willingness to take on hardship and overcome difficulties (Hồng, nursery; Lan Anh, electronics; Lan, construction). This quality could be evaluated by employers during interviews through graduates’ responses to scenarios. According to Davies and Harré (1990), interactive positioning occurs when one positions another individual in storylines. In our study, this form of positioning allows us not only to interpret employers’ expectations of graduate employability but also understand their normative beliefs about how to engage and perform effectively in their disciplinary field as reflected in their discursive pronouncements of what is expected of new graduates. A number of participants (e.g. Huệ, Trinh, Mai and Tú) explained why graduates’ ability to work under pressure is considered as an important attribute that would help graduates become more employable. Participants who worked for manufacturing firms spoke of various pressures confronting an employee, including pressures from above and under their positions. Novice employees in Huệ and Mai’s large-sized companies of over 2500 and over 1000 staff, respectively, faced the pressure of time and workload due to their participation in extensive training after recruitment.

Further, employees encountered constraints related to productivity and emergencies, which often led to their night work. Huệ (electronics manufacturing) added: ‘If they [employees] do not achieve the desired productivity, pressures will accumulate’, which might result in on-going anxieties. In addition, pressures might involve working under unfavourable conditions as in Trinh’s example below:

They are under-staffed. You are a technician, but they need a person who can sit and watch the machines operating in a boiling temperature, you must work in that environment. If you can do that it’s fine, but if you cannot, you will be dismissed. (Trinh, telecommunication)

The above situation requires an employee to be willing to take on hardship and display his/her persistence and diligence. In similar circumstances, Tú (manufacturing) pointed out that approximately 20% of the recruited personnel in her organisation ‘could not endure the pressures’. Trinh (telecommunication) concurred, ‘if they could work for more than three months, it means that they could put up with the pressures.’

### Self-learning and life-long learning skills

Self-learning and life-long learning skills were perceived by most participants in this study as crucial skills that graduates needed to develop to respond to the recent and future changes in the socio-economic context and the competitive demands of the labour market in Vietnam. Barnett (2006) points out that “the situations in which learners are likely to find themselves through the rest of their lives are open-ended” (p. 51). Within the Vietnamese context, scholars (e.g. Tran, 2015; Tran et al., 2014) argue that professional trajectories for Vietnamese graduates are becoming more diversified, dynamic and fluid, creating a pressing demand for life-long self-learning. In the education sector, Education Reform Policy 2020 requires change of textbooks, education objectives and pedagogy (Sơn, banking). In this context, self-learning and life-long learning skills were regarded by participants in this field as essential to enable new teachers to catch up with the fast changes in teaching and learning methods as well as knowledge in the field of education. Teachers, according to Hồng (nursery), needed to keep abreast of new knowledge and methods, including new international developments in their field. In the agriculture sector, likewise, recent signature innovations initiated by the government, including Advanced Technology Agriculture and Agriculture 4.0 put agriculture graduates under the pressure of continuous learning to respond to new professional demands (Nam, agriculture).

Underscoring self-learning and life-long learning skills among graduates, Hồng (nursery) stressed, ‘Self-learning is the foundational skill of all skills’. In relation to this quality, many participating employers emphasised job seekers’ willingness to learn on the job and their ability to understand and pick up new ideas quickly. Some participants even stressed that graduates’ ability to continue learning was more important than their disciplinary knowledge at the time of recruitment. In their perspectives, employees could constantly upgrade their knowledge as their organisations tended to train and re-train new employees after hiring them. The capacity of continuous learning allowed graduates to ‘frequently research from practice and from theory’ (Lan, construction). Huệ’s comment seemed to sum up other participants’ demand for this skill among graduates: ‘They must have the willingness to learn, it means they will get further in their work. That attitude for learning, that sense of progressiveness will help them to improve […]’.

### Foreign language and minority language skills

The study unveils employers’ expectation of candidates’ ability to communicate in foreign languages to meet the growing demands of inter-cultural, multi-lingual working environments. Participants (e.g. Hồng, Tú, Huệ and Hoàn) described the multi-cultural, multi-lingual settings of their organisations where Vietnamese workers and white-collar employees worked under the management of Korean, Japanese and Chinese employers. To be retained by foreign-owned and foreign-invested companies like theirs, Vietnamese graduates were expected to obtain proficient communicative foreign language skills. The interviews with these participants indicated that their businesses were in bad need of graduates who could speak Korean, Japanese and Chinese, the native languages of their bosses. Competence in foreign language skills, according to Hồng (electronics manufacturing), enabled employees to communicate directly with their bosses without the need for interpreters, which could enhance work efficiency. As she emphasised, such language skills helped a candidate stand out in a large number of applicants; and those without foreign language skills were unlikely to be employed by her business. In addition, the ability to use a foreign language such as English is expected in some organisations for professional development purposes:

If they (the employees) know a foreign language, they will access the information more quickly. There are many resources in foreign languages so foreign language ability is a means of using these resources to improve their knowledge. (Hưng, agriculture)

English is important and English resources are available on the Internet. If your English is good, you can improve your expertise. Your skills will decide your success in the future. (Tuấn Anh, telecommunication).

Another important finding related to graduates’ language competence was employers’ expectations of their ability to communicate in local languages such as Thái, Mường or H-mong, the dialects of minority groups living in this region (Nam, agriculture; Lan, construction). Participants working in the construction and agriculture sectors highlighted this requirement because of the real demands from the jobs: graduates were expected to connect with local people ‘to win their hearts and minds’ (Nam). Once recruited, agriculture employees also needed to acquire a local language certificate in order to be promoted to a higher position.

## Discussion

In response to a call for a comprehensive study of graduate employability in Vietnam (Nghia, 2019), the current study provides ample empirical evidence about the new sets of skills and attributes emerging in the Northern mountainous region that are of growing importance but still little researched in the field of graduate employability. Using positioning theory (van Langenhove & Harré, 1999) the study shows the discursive processes concerned the way Vietnamese employers ascribed the duties of graduates. The data have demonstrated a demand for graduates to develop and possess the capacity of flexibility and adaptability if they are to perform their professional duties effectively in the local context. Candidates should be prepared and willing to take on different jobs in a variety of working environments, which helps them expose to varied experiences, accumulate a wide range of skills and knowledge and have greater opportunities to learn on the job.

The findings also present the participating employers’ positioning of self-learning and life-long learning skills as expected employability skills among graduates. According to van Langehove (2017), positioning occurs in a given context with certain sets of rights and duties serving as enabler or inhibitor to one’s action in a particular situation. In the specific context of the Northern mountainous region where there have recently been rapid changes in socio-economic conditions and consequently an increasingly demanding labour market, graduates’ self-learning and life-long learning skills are positioned as a facilitator to enhance their employability. This necessitates tertiary education to help students to master these skills so that they can be more competitive in their job search upon graduation.

Within positioning theory, people’s self-positioning and other positioning are influenced by both structural conditions and individual characteristics and attributes (Barnes, 2004; van Langenhove, 2017). The data illustrate how the participating employers’ positioning of graduates’ foreign language and minority language competence as essential employability skills is shaped by the changing and traditional contexts of the Northern mountainous region. On the one hand, the increasing appearance of inter-cultural, multi-lingual working environments results in foreign language proficiency being highlighted as a highly valued graduate attribute. On the other hand, due to the large concentration of ethnic minorities in this region, competence in local minority languages is regarded as crucial for the success of potential employees in the fields that require close communication with local residents such as agriculture and forestry. The findings, therefore, suggest that local universities explore and address the language needs of the regional labour market to increase their graduates’ competitiveness and employability.

Graduates in the Northern mountainous region are, thus, positioned with the expected duties associated with being flexible, adaptable and engaged in continuous self-learning, possessing foreign language and minority language competence as well as demonstrating resilience and empathy for the locals and local environment. Positioning theory (van Langenhove & Harré, 1999) helps us to explore the dynamics of the intersecting forces that influence what constitutes graduate employability in the local labour market. Within this study of the Northern mountainous region of Vietnam, these forces are comprised of not only the supply and demand factors, professional field of work and workplace culture but also, the distinctive demographic conditions of the region. Employers ascribed the expected duties and positioned the attributes of graduates within this intersection of multiple forces. This study sheds light on the ways graduate employability is context-situated and characterised by the demographic conditions and cultural norms of the local region. The study, thus, lends support for developing our understandings of how employability can be contextualised. The findings of the study, therefore, highlight a critical need to closely look at the notion of ‘employability in context’ in understanding what is expected of our graduates in the labour force, especially in contexts that are not commonly discussed in the current literature on graduate employability, such as disadvantaged, remote and mountainous regions.

In the last quarter of 2020, of 53,950 million workers in Vietnam, 67.33% were located in the rural region (MOLISA, 2020). Yet, this research found that it is challenging for employers in rural and mountainous areas not only to find candidates who possess relevant capabilities but also to retain them. Employers reported that graduates from urban regions appear to be unwilling to come to work in the Northern mountainous region. Graduates from urban universities who are employed in regional and rural industries often desire to relocate to the city. On the other hand, many graduates from universities in rural and mountainous regions face challenges in finding appropriate employment in line with their education due to the disconnect between tertiary education and labour market needs (ISEE, 2011; Tran et al., 2014). There is a dual concern that the rural mountainous region is experiencing brain drain and unemployment or under-employment at the same time. Strategic incentives to supplement the unfavourable living conditions to attract and retain capable graduates are needed. Simultaneously, it is crucial to support curriculum enhancement and innovation to improve the alignment between tertiary education and labour market needs.

### Implications of the study

The study has significant implications for universities and related stakeholders, especially in the rural and mountainous regions, to provide graduates with conducive conditions to develop ‘employability in context’ rather than only technical and generic employability skills. The development of ‘employability in context’ is subject to a coordinated approach involving key stakeholders such as local employers in the region and organisations, curriculum design and career support services that should be tailored to meet students’ needs and career orientations. This finding raises the awareness of the importance of developing the in-context employability attributes in the higher education programme and the connection between universities with local employers and communities. Organisation of activities to facilitate students’ participation in the local community and community-based activities in the rural region to develop context-situated work capabilities and local engagement is highly recommended.

The study calls for a critical reform in higher education curriculum and career guidance field which focuses on not only developing graduates’ generic professional skills but also the ‘local’ contextualised attributes, especially adaptability, flexibility, resilience, commitment and continuous learning, empathy, devotion and commitment to the development of local context. Although these in-context attributes are required in a wide range of industries such as education, agriculture, construction, telecommunication and electronics in both the public and private sectors as the result of the distinctive demographic conditions of the region, they are not uniform. More specifically, the extent to which the skills are demanded by employers depends on the nature of the work in this context. For example, while local language competency is expected in all sectors, it is more crucial for employees working in agriculture and telecommunication as they must work closely with the local people who cannot speak Kinh, the official language in Vietnam. This suggests the important role of career counselling services to help graduates keep abreast of the employability attributes demanded in the dynamic and changing labour markets so that they can prioritise building these capacities earlier in their university experiences.

### Limitations of the study

The study is restricted in its scope, interviewing 40 employers in a limited number of sectors in the region. Therefore, further research based on a large-scale survey of employers across a wide range of sectors would be helpful to capture the complexities and dynamics of the labour market demands and employer expectations of graduate employability in important but under-represented contexts, especially in disadvantaged, remote and mountainous regions. Another limitation of the study is that it does not look into the extent to which the development of contextualised employability attributes has been incorporated into university programmes, from the perspectives of university staff.

## Conclusion

This study provides the empirical base to introduce the concept of ‘employability in context’. In this research, the employability for graduates in the Northern mountainous region is context-situated with the expected core attributes as being engaged in continuous self-learning and adaptive to the geographical conditions and traditions of the rural area, as well as demonstrating resilience in working in disadvantaged areas and empathy for the locals and local mountainous environment. The findings, therefore, support the need for employability to considered on a contextual basis, taking into account demographic conditions and cultural norms of the local region, especially regions that are under-represented in the literature such as the Northern mountainous region in Vietnam. The study underscores the importance to look beyond universal employability components to understand graduate employability in marginalised contexts in Vietnam and across different parts of the world.
